# Supplementary material for: Macroscopic waves, biological clocks and morphogenesis driven by light in a giant unicellular green alga
Source: Nat Commun. 2023 Oct 4;14:6204. doi: 10.1038/s41467-023-41813-6 (PMC10550971; doi:10.1038/s41467-023-41813-6)
Supplement: Supplementary file 1 — Supplementary Information [file 41467_2023_41813_MOESM1_ESM.pdf]

# Supplementary Information

## Macroscopic waves, biological clocks and morphogenesis driven by light in a giant unicellular green alga

Eldad Afik,[1,2*] Toni J.B. Liu,[1] Elliot M. Meyerowitz[1,2*]

[1]Division of Biology and Biological Engineering, California Institute of Technology, 1200 E California Blvd., Pasadena, CA 91125, USA
[2]Howard Hughes Medical Institute

*Correspondence to eldad.afik@gmail.com, meyerow@caltech.edu

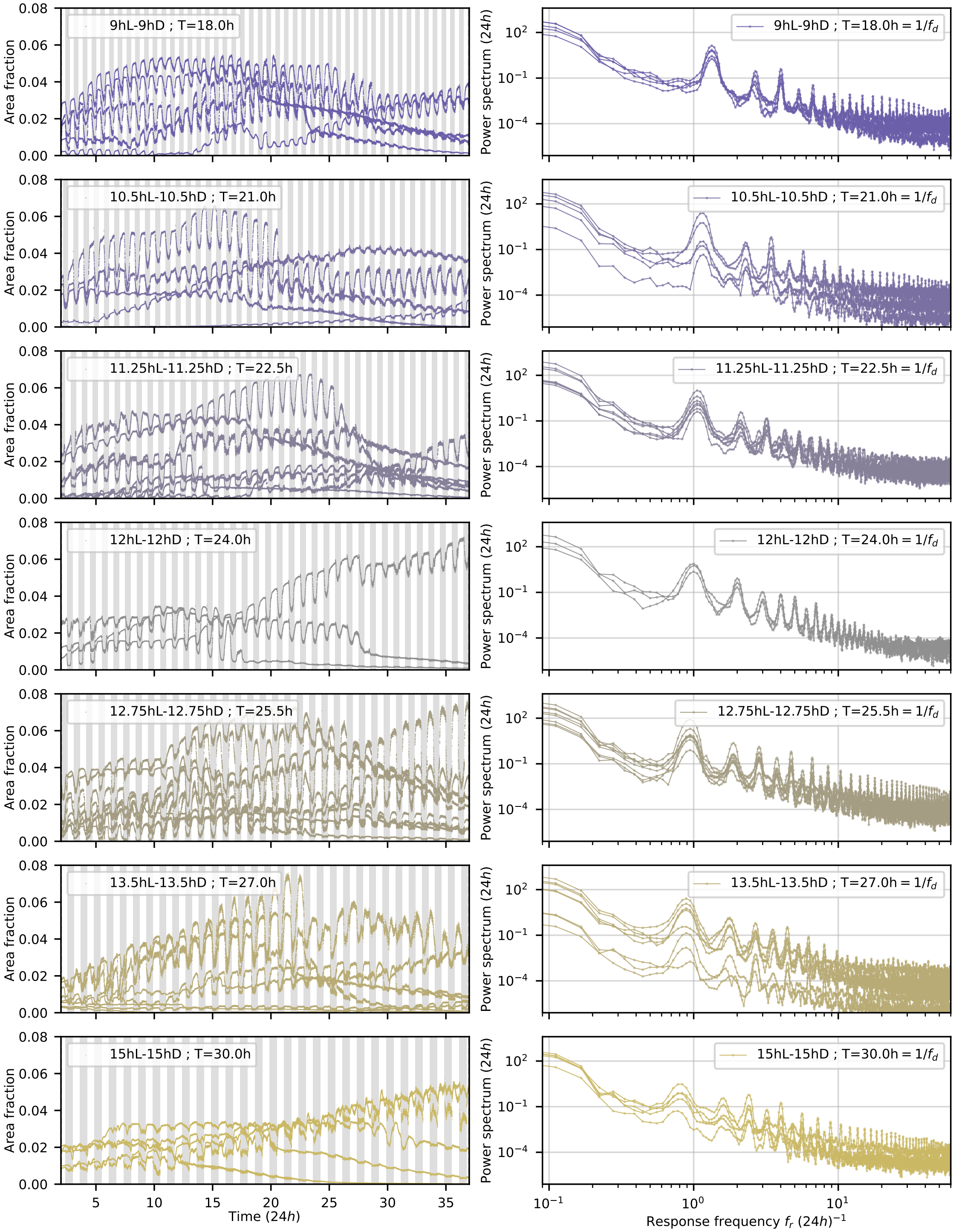


**Supplementary Fig. 1**: **Time series and corresponding power spectra under driving illumination periods within 18h to 30h.** Area fraction corresponds to the measured green area within a whole well, normalised by the area of the well. Each curve corresponds to a distinct sample. Response fundamental frequencies $f_{r,0}$ inferred from the power spectra on the right are presented in Fig. 2d. Averages over power spectra curves, grouped by driving illumination frequencies, $f_d = 1/T$, are shown in Fig. 2c. The data in Fig. 2a,b are presented here again for comparison, labeled "12hL-12hD ; T=24.0h".

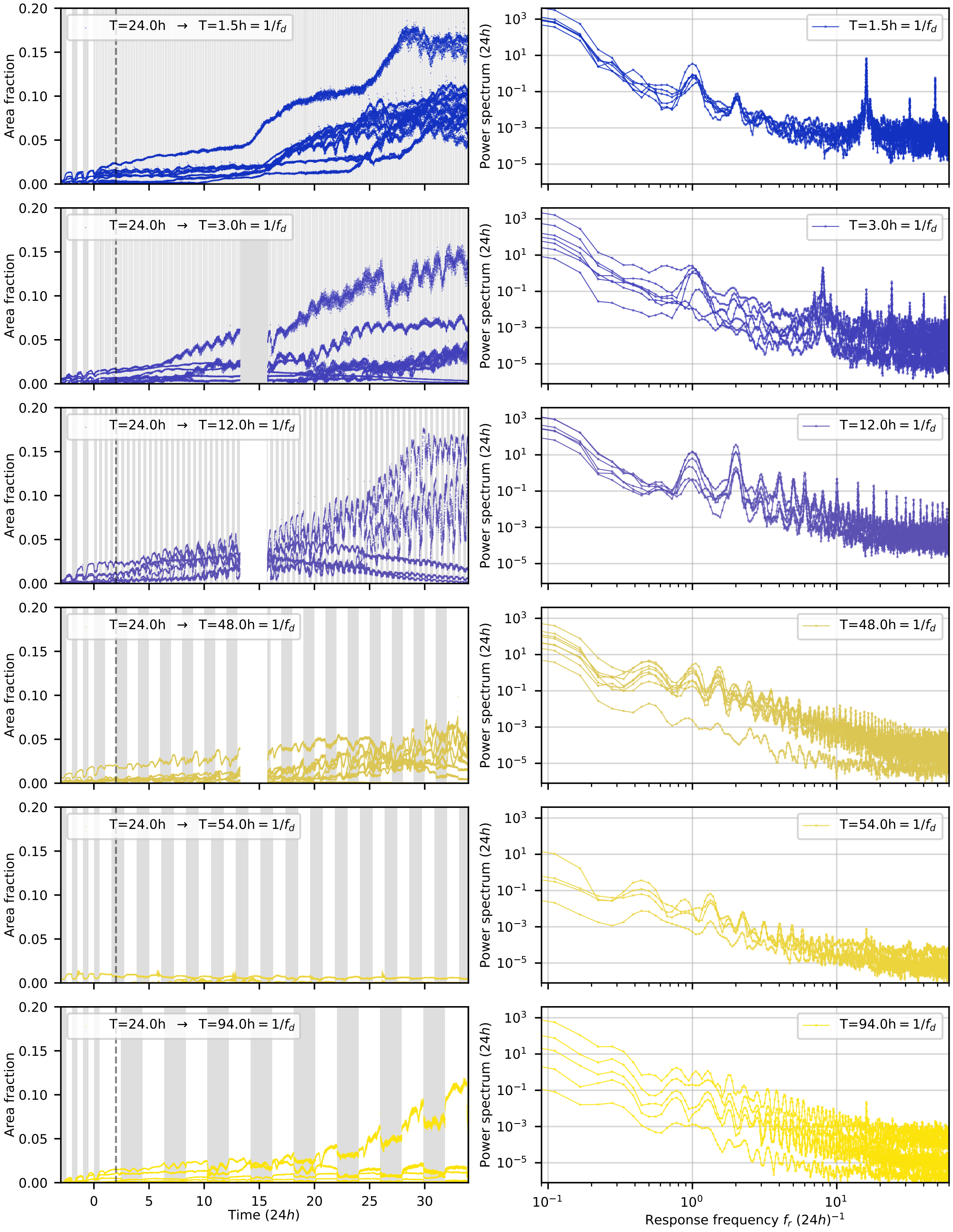


**Supplementary Fig. 2**: **Time series and corresponding power spectra under driving periods far from 24h.** Averages over the power spectra curves on the right, grouped by driving illumination frequencies $f_d = 1/T$, are presented in Fig. 3c as heatmaps. Examples from the T=3h and T=48h panels on the left are highlighted in Fig. 3a,b. Power spectra are inferred from intervals starting two days after the transition from 12hL-12hD to the new driving illumination protocol, marked by the dashed vertical line on the time series.

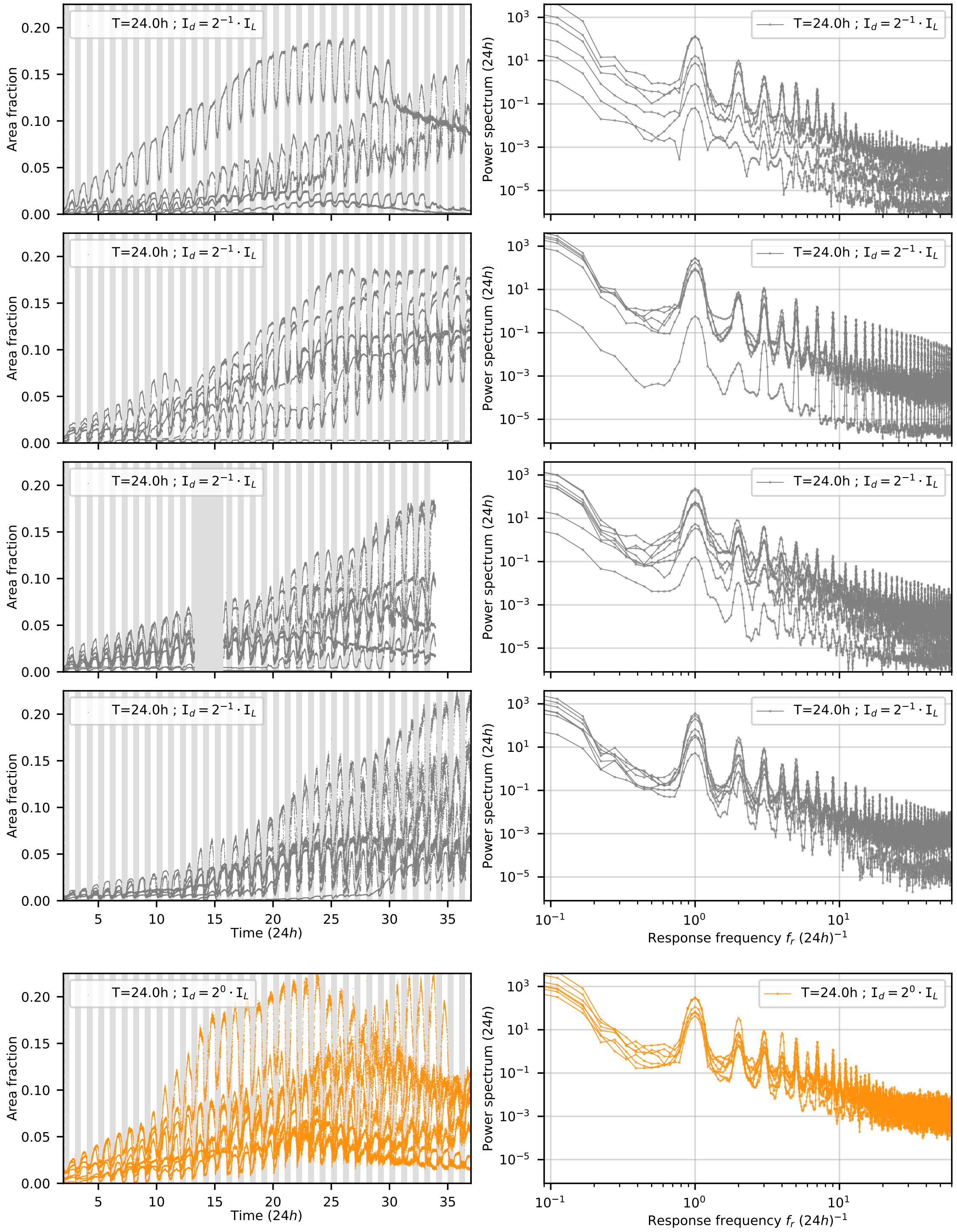


**Supplementary Fig. 3**: **Additional time series and corresponding power spectra under driving period T=24h.** Panels are grouped by culture dish. Averaging over the power spectra curves corresponding to T=24h $\mathtt{I}_d = 2^{-1} \cdot \mathtt{I}_\mathtt{L}$, in grey (top four panels), together with T=24h in Supplementary Fig. 1, results in the heatmap column labeled $f_d = 1/24h$ in Fig. 3c. For comparison, samples subject to T=24h $\mathtt{I}_d = 2^0 \cdot \mathtt{I}_\mathtt{L}$ are presented in orange (bottom panel).

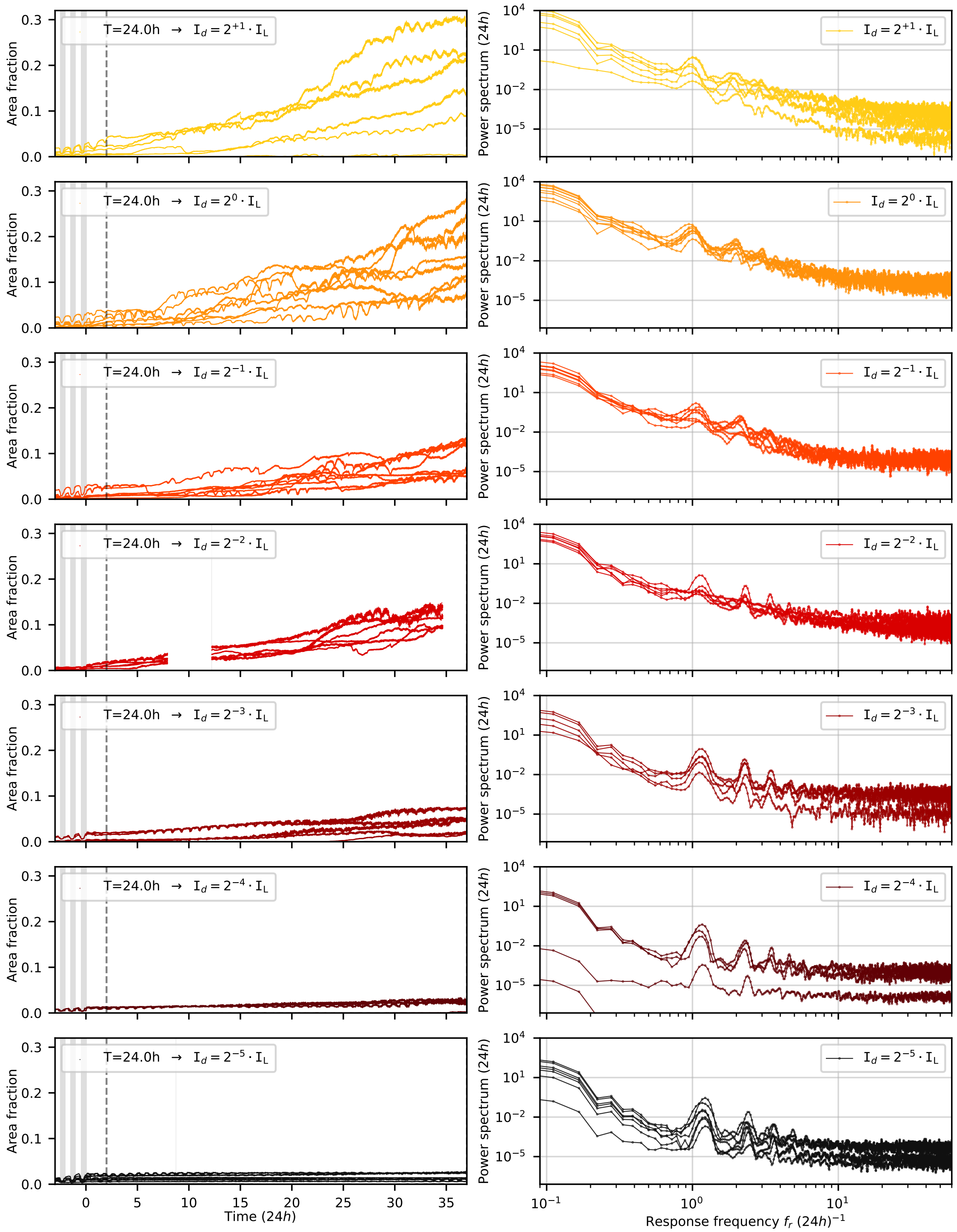

**Supplementary Fig. 4**: **Time series and corresponding power spectra under constant illumination intensities within $2^{-5} \cdot \mathtt{I}_\mathrm{L}$ to $2^{+1} \cdot \mathtt{I}_\mathrm{L}$.** Response fundamental frequencies $f_{r,0}$ inferred from the power spectra on the right are presented in Fig. 4c. Averages over power spectra curves, grouped by driving illumination $\mathtt{I}_d$, are shown in Fig. 4d. Examples from the $\mathtt{I}_d = 2^{-5} \cdot \mathtt{I}_\mathrm{L}$ and $\mathtt{I}_d = 2 \cdot \mathtt{I}_\mathrm{L}$ panels on the left are highlighted in Fig. 4a,b. Power spectra are inferred from intervals starting two days after the transition from 12hL-12hD to the new driving illumination protocol, marked by the dashed vertical line on the time series.

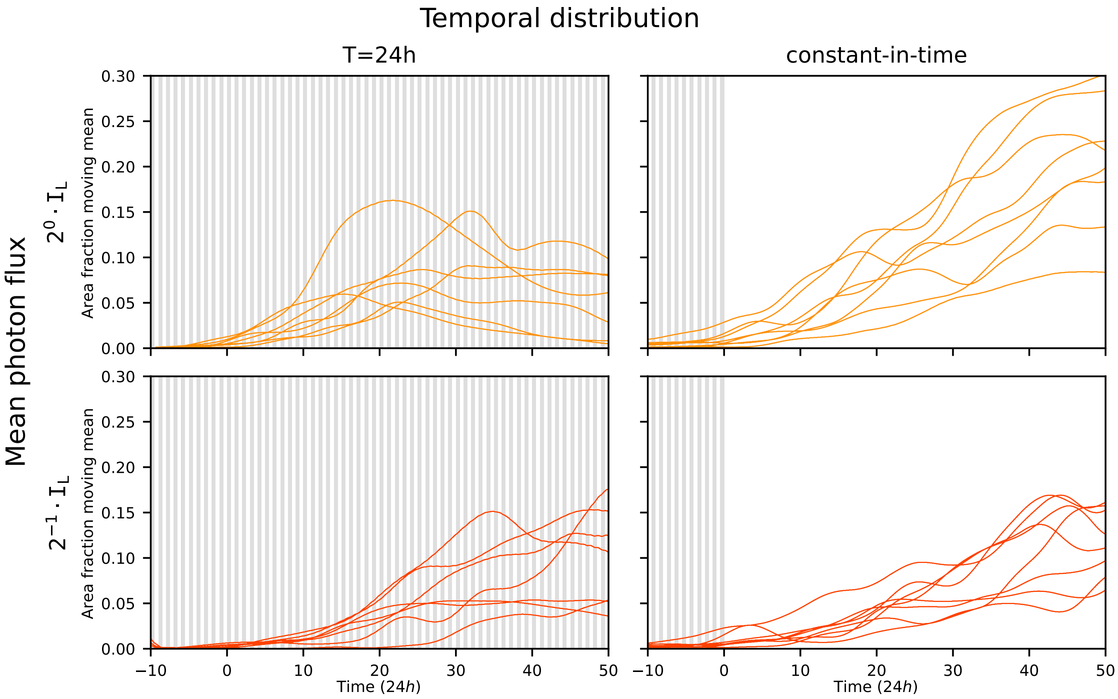


**Supplementary Fig. 5: Apparent area growth curves comparing mean photon flux levels $2^0 \cdot \mathtt{I}_\mathtt{L}$ and $2^{-1} \cdot \mathtt{I}_\mathtt{L}$, at T=24h and constant illumination.** Growth curves inferred from time-series by moving averages. The constant-in-time column shows data presented in Fig. 4e, plotted again here for comparison with T=24h illumination. Snapshots on day 25 of three samples from each of the above panels are presented in Fig. 5.
