# Supplementary material for: Macroscopic waves, biological clocks and morphogenesis driven by light in a giant unicellular green alga
Source: Nat Commun. 2023 Oct 4;14:6204. doi: 10.1038/s41467-023-41813-6 (PMC10550971; doi:10.1038/s41467-023-41813-6)
Supplement: Supplementary file 3 — Description of Additional Supplementary Files [file 41467_2023_41813_MOESM3_ESM.pdf]

Description of Additional Supplementary Files

Supplementary Movie 1. Time-lapse imaging of algal samples regenerating under 12hL-12hD, T=24h cycles. One month of regeneration under 12h Light - 12h Dark; 3 seconds correspond to 24h; 3 wells, 3cm x 4cm each. The waves of greenness show synchronisation within individual cells, and among them.
