# Supplementary material for: Macroscopic waves, biological clocks and morphogenesis driven by light in a giant unicellular green alga
Source: Nat Commun. 2023 Oct 4;14:6204. doi: 10.1038/s41467-023-41813-6 (PMC10550971; doi:10.1038/s41467-023-41813-6)
Supplement: Supplementary file 5 — Reporting Summary [file 41467_2023_41813_MOESM5_ESM.pdf]

nature portfolio

Corresponding author(s): Elliot M. Meyerowitz
Eldad Afik

Last updated by author(s): Sep 10, 2023

Reporting Summary

Nature Portfolio wishes to improve the reproducibility of the work that we publish. This form provides structure for consistency and transparency in reporting. For further information on Nature Portfolio policies, see our Editorial Policies and the Editorial Policy Checklist.

Statistics

For all statistical analyses, confirm that the following items are present in the figure legend, table legend, main text, or Methods section.

n/a	Confirmed	
☒	☐	The exact sample size (*n*) for each experimental group/condition, given as a discrete number and unit of measurement
☒	☐	A statement on whether measurements were taken from distinct samples or whether the same sample was measured repeatedly
☒	☐	The statistical test(s) used AND whether they are one- or two-sided<br>*Only common tests should be described solely by name; describe more complex techniques in the Methods section.*
☒	☐	A description of all covariates tested
☒	☐	A description of any assumptions or corrections, such as tests of normality and adjustment for multiple comparisons
☒	☐	A full description of the statistical parameters including central tendency (e.g. means) or other basic estimates (e.g. regression coefficient) AND variation (e.g. standard deviation) or associated estimates of uncertainty (e.g. confidence intervals)
☒	☐	For null hypothesis testing, the test statistic (e.g. *F*, *t*, *r*) with confidence intervals, effect sizes, degrees of freedom and *P* value noted<br>*Give P values as exact values whenever suitable.*
☒	☐	For Bayesian analysis, information on the choice of priors and Markov chain Monte Carlo settings
☒	☐	For hierarchical and complex designs, identification of the appropriate level for tests and full reporting of outcomes
☒	☐	Estimates of effect sizes (e.g. Cohen's *d*, Pearson's *r*), indicating how they were calculated

Our web collection on statistics for biologists contains articles on many of the points above.

Software and code

Policy information about availability of computer code

Data collection

Raspbian GNU/Linux 10 (buster)
https://github.com/silvanmelchior/RPi_Cam_Web_Interface V6.6.13
Python 3.7.3
IPython 5.8.0

http://abyz.me.uk/rpi/pigpio/python.html V71
https://gpiozero.readthedocs.io/en/stable/ v1.5.1

Data analysis

All programming and computer aided analysis has been done using open-source projects, primarily tools from the Scientific Python ecosystem.

conda 4.14.0
mamba (conda 22.11.1)

Python implementation: CPython
Python version : 3.7.12
IPython version : 7.33.0

scipy : 1.7.3

numpy : 1.21.6
xarray: 0.20.2
pandas: 1.3.5

PIL : 8.4.0
skimage : 0.19.3
dask : 2022.2.0
dask_image: 2021.12.0

zarr : 2.12.0
pyarrow: 9.0.0

napari: 0.4.14

jupyterlab : 3.4.6
tqdm : 4.64.1
ipywidgets : 8.0.2
jupyterlab_widgets: 3.0.3

matplotlib : 3.7.1

hvplot : 0.8.1
holoviews : 1.15.1
bokeh : 2.4.3
datashader: 0.14.2

Custom computer codes used to analyse the results reported in the manuscript are available in the figshare repository, 28210.6084/m9.figshare.23797020,²⁷ and from the corresponding authors on reasonable request.

For manuscripts utilizing custom algorithms or software that are central to the research but not yet described in published literature, software must be made available to editors and reviewers. We strongly encourage code deposition in a community repository (e.g. GitHub). See the Nature Portfolio guidelines for submitting code & software for further information.

Data

Policy information about availability of data

All manuscripts must include a data availability statement. This statement should provide the following information, where applicable:

- Accession codes, unique identifiers, or web links for publicly available datasets
- A description of any restrictions on data availability
- For clinical datasets or third party data, please ensure that the statement adheres to our policy

The datasets generated and analysed during the current study are available in the figshare repository, 10.6084/m9.figshare.23797020 :
Afik, E. & Meyerowitz, E. M. Dynamical states of self-organised waves in a giant unicellular green alga. figshare. Dataset. (2023). https://doi.org/10.6084/m9.figshare.23797020

Human research participants

Policy information about studies involving human research participants and Sex and Gender in Research.

Reporting on sex and gender	N/A
Population characteristics	N/A
Recruitment	N/A
Ethics oversight	N/A

Note that full information on the approval of the study protocol must also be provided in the manuscript.

Field-specific reporting

Please select the one below that is the best fit for your research. If you are not sure, read the appropriate sections before making your selection.

☒ Life sciences ☐ Behavioural & social sciences ☐ Ecological, evolutionary & environmental sciences

For a reference copy of the document with all sections, see nature.com/documents/nr-reporting-summary-flat.pdf

Life sciences study design

All studies must disclose on these points even when the disclosure is negative.

Sample size

Experiments were designed to result in at least 3 samples per illumination protocol.
Illumination protocols were applied to at least one culture dish, each consisting of 8 wells.

Time-series filtering procedure is described in the manuscript in the Methods Section, and copied below under "Data Exclusions".

The resulting sample sizes per illumination protocol are listed below, organized by the manuscript figure these contributed to.
A degree of sample dispersion within illumination protocols can be appreciated from the "dot-plots" in Fig. 2d and Fig. 4c

Fig. 2 and Fig. 3
============
T=18h 5
T=21h 5
T=22.5h 7
T=24h 3
T=25.h 8
T=27h 8
T=30h 5

Fig. 3
=====
T=1.5h 6
T=3h 7
T=12h 6
T=24h 27 (4 culture dishes from 3 distinct experiments)
T=48h 8
T=54h 4
T=94h 5

Fig. 4
=====
2^1 6
2^0 8
2^{-1} 8
2^{-2} 6
2^{-3} 6
2^{-4} 5
2^{-5} 7

Data exclusions

From the Methods Section in the manuscript:

Post-processing of the time series consisted of outlier detection and replacement. Outliers have been defined as those exceeding 75% of the well, or lying outside the 1.5 x Interquartile-Range of a rolling window spanning 1h40m. Where there were no more than three consecutive outliers, these have been replaced by linear interpolation.

Following the above post-processing procedure for outlier detection and replacement, time series consisting of more than 2% unreplaced outliers within these intervals were excluded from this report.

Any remaining detected outliers were replaced by forward and backwards propagation of nearest valid data.

Replication

Measures for data reproducibility are detailed in the Methods section and consist of the Cut & Regenerate procedure, as well as controlled temperature and computerized illumination conditions. All attempts at replication have been successful so far, with the exception of two experiments which did not show regeneration. These two showed signs of contamination in the culture medium, and are not included in this study.

Randomization

All culture dishes were prepared following the same Cut&Regen protocol. Illumination protocols were assigned randomly within each batch of illumination experiments.

Blinding

All culture dishes were prepared following the same Cut&Regen protocol. Filtering follows the procedure detailed under Data Exclusions, designed to be protocol agnostic.

Reporting for specific materials, systems and methods

We require information from authors about some types of materials, experimental systems and methods used in many studies. Here, indicate whether each material, system or method listed is relevant to your study. If you are not sure if a list item applies to your research, read the appropriate section before selecting a response.

Materials & experimental systems

n/a	Involved in the study
☒	☐ Antibodies
☒	☐ Eukaryotic cell lines
☒	☐ Palaeontology and archaeology
☐	☒ Animals and other organisms
☒	☐ Clinical data
☒	☐ Dual use research of concern

Methods

n/a	Involved in the study
☒	☐ ChIP-seq
☒	☐ Flow cytometry
☒	☐ MRI-based neuroimaging

Animals and other research organisms

Policy information about studies involving animals; ARRIVE guidelines recommended for reporting animal research, and Sex and Gender in Research

Laboratory animals	The reported study did not involve laboratory animals.
Wild animals	The reported study did not involve wild animals.
Reporting on sex	*Indicate if findings apply to only one sex; describe whether sex was considered in study design, methods used for assigning sex. Provide data disaggregated for sex where this information has been collected in the source data as appropriate; provide overall numbers in this Reporting Summary. Please state if this information has not been collected. Report sex-based analyses where performed, justify reasons for lack of sex-based analysis.*
Field-collected samples	The reported study did not involve samples collected from field.
Ethics oversight	No ethical approval or guidance was necessary for the reported study.

Note that full information on the approval of the study protocol must also be provided in the manuscript.
